# Development and validation of an LC-MS/MS method for determination of 8-iso-prostaglandin f2 Alpha in human saliva

**DOI:** 10.5937/jomb0-33556

**Published:** 2022-10-15

**Authors:** Zlatina Tomova, Desislav Tomov, Angelina Vlahova, Veneta Chaova-Gizdakova, Lyubka Yoanidu, Dobrin Svinarov

**Affiliations:** 1 Medical University of Plovdiv, Faculty of Dental Medicine, Department of Prosthetic Dental Medicine, Plovdiv, Bulgaria; 2 Medical University of Plovdiv, Faculty of Pharmacy, Department of Bioorganic Chemistry, Plovdiv, Bulgaria; 3 Research Institute at Medical University of Plovdiv, Plovdiv, Bulgaria; 4 Medical University of Sofia, Faculty of Medicine, Clinical Laboratory & Clinical Pharmacology, UMBAL Alexandrovska, Sofia, Bulgaria

**Keywords:** metal alloy, 8-isoPGF2a, LC-MS/MS, saliva, SALLE, metalna legura, 8-isoPGF2a, LC-MS/MS, pljuvačka, SALLE

## Abstract

**Background:**

Increased formation of reactive oxygen species may be caused by the ion release of the metal alloys used in prosthetic dental restorations due to the corrosion process. As products of lipid peroxidation, isoprostanes can be used as a marker for oxidative stress in the body. There are two significant advantages of using isoprostanes as an oxidative stress marker - presence in all fluids in the body and low reactivity. Saliva provides noninvasive, painless, and cost-effective sample collection and can be used as an alternative testing medium of blood and urine.

**Methods:**

This study presents the development and validation of a sample LC-MS/MS method to quantify 8-isoprostaglandin F2-a in human saliva using salt-out assisted liquid-liquid extraction (SALLE).

**Results:**

The selected sample preparation procedure optimized chromatographic separation and mass detection provided high recovery and sensitivity of the analysis. The calibration curve was obtained in the predefined range 25-329 ng/L with R2 larger than 0.995. Normalized matrix varied between 89.7 % and 113.5%. The method showed sufficient accuracy and precision - accuracy in the range 89.7 %-113.9 %, and precision between 2.3% and 5.4%.

**Conclusions:**

The proposed method is validated according to current EMA/FDA industrial guidance for bioanalysis and offers an appropriate level of sensitivity and sufficient accuracy and precision.

## Abstract (sr)

**Uvod:**

Povećano formiranje reaktivnih vrsta kiseonika može biti uzrokovano oslobađanjem jona iz metalnih legura koje se koriste u zubnim protezama, a usled procesa korozije. Kao rezultat peroksidacije lipida, izoprostani se mogu koristiti kao marker oksidativnog stresa u telu. Postoje dve značajne prednosti upotrebe izoprostana kao markera oksidativnog stresa - prisustvo u svim tečnostima u telu i niska reaktivnost. Pljuvačka omogućava neinvazivno, bezbolno i isplativo uzimanje uzoraka i može se koristiti kao alternativni medijum za testiranje (alternativa testovima krvi i urina).

**Metode:**

Ova studija predstavlja razvoj i validaciju uzorka LC-MS/MS metode za kvantifikaciju 8-izoprostaglandina F2-a u ljudskoj pljuvački korišćenjem tečno-tečne ekstrakcije koja je potpomognuta ispoljavanjem (SALLE).

**Rezultati:**

Odabrana procedura pripreme uzorka je optimizovala hromatografsko odvajanje a detekcija mase je obezbedila visok povraćaj i osetljivost analize. Kalibraciona kriva je dobijena u unapred definisanom opsegu 25-329 ng/L sa R2 većim od 0,995. Normalizovana matrica je varirala između 89,7 % i 113,5 %. Metoda je pokazala dovoljnu tačnost i preciznost - tačnost u rasponu od 89,7 %-113,9 %, a preciznost između 2,3 % i 5,4 %.

**Zaključak:**

Predložena metoda je validirana u skladu sa važećim uputstvima EMA/FDA za bioanalizu i nudi odgovaraju}i nivo osetljivosti i dovoljnu tačnost i preciznost.

## Introduction

For the production of metal-ceramic prosthetic restorations, two main groups of base metal dental alloys - cobalt-chromium and nickel-chromium are widely used [1]. To implement the innovative CAD/CAM technologies in dental medicine, powder metal alloys for selective laser melting are developed [2]. The most important feature of a dental alloy for its biological safety is its tendency for corrosion. Corrosion resistance depends on the content of the alloy, the method of its production, and the method of producing the metal coping of the PFM restoration [3] [4] [5]. As a result of the corrosion process, ion emission appears from the metal surface. The different metal ions may cause dose-dependent cytopathological effects in specific cell types [6]. Metals are potentially toxic in their ionic forms and may cause increased production of reactive oxygen species [7] [8]. According to Akbar et al. [9], 2011, ion emission initiates cell apoptosis which leads to a decrease in lymphocyte proliferation.

Reactive oxygen species (ROS) are a part of the normal metabolism of the human body, of its signaling and defensive systems. Their production and neutralization are in equilibrium [10]. Because of their high reactivity, each disturbance of this balance leads to oxidative stress with damage of biomolecules - lipids, proteins, nucleus acids. The products of interaction between ROS and other biomolecules can be used to evaluate oxidative stress levels. Free radical damage of lipids leads to disturbance of cell membranes' structure and function and to forming potentially mutagenic and carcinogenic products. It may also cause cell death by apoptosis or autophagy. Unlike prostaglandins, which are formed from the free arachidonic acid under the action of cyclooxygenases (COX), isoprostanes are mainly a result of non-enzymatic peroxidation of arachidonic acid and its esters, which are part of cell membranes [11]. The release of isoprostanes from membrane structures is influenced by phospholipases and platelet-activating factor - acetylhydrolase (PAF-AH) [12]. Lipid peroxidation products may be used as a marker for oxidative stress in the body. There are two big advantages of using isoprostanes as an oxidative stress marker - presence in all fluids in the body and low reactivity. Furthermore, their local concentration may be used to evaluate and observe the specific area of the body [13].

Saliva can be used as an alternative testing medium of blood and urine [14]. It provides non-invasive, painless, and cost-effective sample collection [15]. Levels of some substances in saliva like uric acid and creatinine are measurable and correlated with their plasma levels [16].

Although saliva is not a widely utilized biological fluid for laboratory analysis, the number of studies using it has increased. There are studies using spetrophotometric methods for measuring glucose levels in the body [17]. Enzyme-linked immunosorbent assays (ELISA) are applied for the detection of SARS-CoV-2 antibodies in saliva [18], antibodies against other viruses (HIV, poliovirus), salivatory cytokine levels [19], isoprostanes [20]. Liquid chromatography-tandem mass spectrometry (LC-MS/MS) methods are used for analyzing levels of phenytoin [21], risperidone [22], theophylline [23], estrogens, cortisol, melatonin [24], uric acid, and creatinine [25]. Inductively coupled plasma-mass spectrometry (ICP-MS) is used to determine trace elements and heavy metals in saliva [26].

This study aims to develop and validate a method of detecting 8-isoprostaglandin F2-α in human saliva using liquid chromatography-tandem mass spectrometry (LC-MS/MS).

## Materials and methods

### Chemicals and reagents

LC-MS grade methanol (MeOH), LC-MS grade acetonitrile, and HPLC grade ethyl acetate were provided by Honeywell (Charlotte, USA); formic acid, hexane, 1-butanol, sodium chloride, magnesium sulfate, and zinc sulfate heptahydrate were purchased from Sigma-Aldrich (Steinheim, Germany); chloroform was provided by VWR Chemicals BDH (Avantor, USA); analytical standards of 8-isoPGF2α and 8-isoPGF2α-d4 were purchased from Cayman Chemicals (Ann Arbor, MI, USA). Deionized water was produced in the laboratory with the ELGA Veolia Chorus system (ELGA Lab Water, UK). The stock solution of 8-isoPGF2α and 8-isoPGF2α-d4 (IS) were prepared in methanol and stored at -20°C.

### Preparation of working and calibration solutions and quality control samples

Working solutions were made in 50% methanol with concentrations of 1.33, 2, 2.4, 3.6, 5.4, 8.1, 16.2, and 32.4 μg/L for the preparation of the calibration solutions, and 2, 2.5, 5, and 25 μg/L-for preparation of the lower limit of quantification and quality control samples. Saliva from young, healthy subjects without metal or metal-ceramic prosthetic restorations, who had signed informed consent to participate in the project, was used for the preparation of calibration solutions and control samples as follows: 10 μL of the respective working solution was added to 990 μL of saliva pool and vortexed gently for 5 min, and frozen at -20°C. Resulting concentrations added were 13.3, 20, 24, 36, 54, 81, 161 and 324 ng/L; for control samples -20, 25, 50 and 250 ng/L.

### Sample collection

Saliva samples were taken by spitting into a plastic container 2-3 hours after oral hygiene procedures between 9.00 and 12.00 pm. The saliva collection was done in the dental office without external irritants like visual, olfactory, or acoustic stimuli. Patients were asked to sit on the dental chair comfortably with their heads slightly inclined forward. After rinsing the oral cavity with distilled water, patients spat the saliva gathered in the mouth in the container instead of swallowing it. The collected saliva was centrifuged at 6500 rpm for 10 minutes to remove debris and frozen at -70°C until analysis.

### Sample preparation

In a 5 mL sample tube, 1 mL of a freshly thawed saliva sample and 100 μL of internal standard solution were added and gently mixed for 5 min. Anhydrous NaCl (0.5 g) was added, and the sample was vortexed for 5 min. 2 mL of extraction solution consisting of ethyl ethanoate was added and vortexed for another 5 min. The sample was centrifuged (10 min, 6500 rpm), and the supernatant was transferred in a 2 mL PP tube for evaporation. The extraction steps were repeated twice, and the collected supernatant was evaporated under a stream of nitrogen at 45°C. The dry residue was reconstituted with 50% methanol and injected for analysis.

### Liquid chromatographic and mass spectrometric conditions

All measurements were carried out using the LC system Dionex Ultimate 3000, consisting of a quaternary pump, an autosampler, and a thermostat for chromatographic columns, connected to TSQ Quantum Access Max triple quadrupole mass spectrometer (Thermo Fisher Scientific, MA, USA). Chromatographic separation was performed with gradient elution on core-shell Accucore^™^ RP-MS 100 x 2.1 mm, 2.6 μm particles analytical column (Thermo Fisher Scientific, MA, USA). The column was tempered at 25°C. Mobile phases B and D consisted of 0.1% formic acid in methanol and 0.1% formic acid in methanol/water (55:45, v/v). Chromatographic separation was achieved by gradient elution: 0-6 min 20% B, 6-8 min 20-95% B, 8-15 min 95-100% B, 15-16 min 20% B, and held at 20% B until the end of the run. The total run time was 30 min. Thermo Xcalibur^™^ (V 2.2 SP1.48) was used to control the system and data acquisition and processing.

For analyte detection, heated electrospray ionization (HESI) in negative ionization mode was used with optimized parameters – pray voltage – 3000; vaporizer temperature 280°C; sheath gas, 40 arbitrary units; capillary temperature 275°C. Deprotonated analyte molecules and internal standards were used as precursor ions for selective reaction monitoring (SRM) with the transition of m/z 353 → 193 for 8- isoPGF2α and 357 → 197 for 8-isoPGF2α-d4. Argon was used as collision gas; collision energy was 28 V. Calculation of concentration was performed by the method of background subtraction.

### Statistical data analysis

Microsoft Excel program was applied for statistical processing of received data.

### Method validation

**Figure 1:**
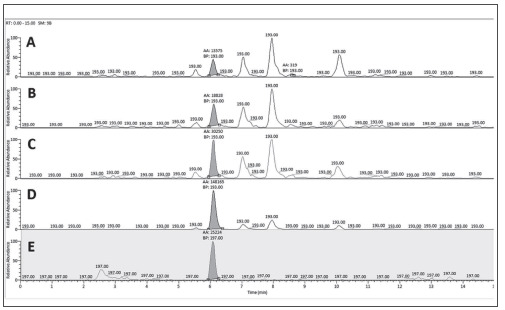
SRM chromatograms obtained by analysis of control samples at four concentration levels – 25 ng/L (A), 30 ng/L (B), 55 ng/L (C), 255 ng/L (D), and internal standard (E).

The calibration curve was plotted using the dependence of the ratio of the peak area of 8-isoPGF2α to the peak area of internal standard (8-isoPGF2α-d4) and concentration (Figure 1). The selectivity of the method was assessed by six different saliva samples with standard addition techniques at two concentration levels. The predefined normalized matrix effect was within 85-115%. The intra-day accuracy and precision were assessed by analyzing five sample replicates at three concentration levels (30, 55, and 255 ng/L) and at a low limit of quantification level (25 ng/L). The between-day accuracy and precision were assessed by analyzing two sample replicates on four different days at the same concentration levels. Imprecision and inaccuracy, intra-and between days, should be in the range of 15% for control samples and in the range of 20% for the low limit of quantification samples. The stability of spiked saliva samples was determined in different storage conditions - within three freeze-thaw cycles and for 12 and 24 h at 4°C. The validation procedure was designed according to current EMA/FDA industrial guidance for bioanalysis via LC-MS/MS [27] [28].

## Results and discussion

**Figure 2:**
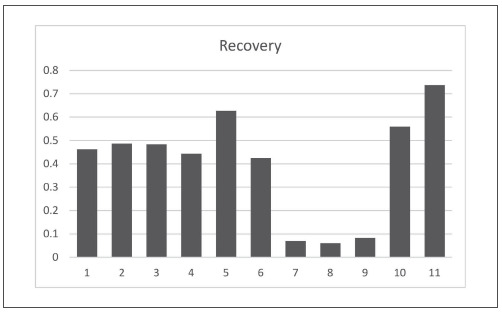
Calculated recovery for 11 different sample preparation procedures.

Saliva is aqueous biological media secreted by the large and the minor salivary glands, and although it consists of above 95% water, it also contains numerous organic and inorganic components with different polarity - ions, uric acid, glucose, cholesterol, fatty acid, triglycerides, lipids, glycolipids, steroid hormones, proteins, immunoglobulins, enzymes, peroxidase, lactoferrin, etc. The main obstacle to the development of this method was the low level of the analyte in the saliva samples. Because of this, it was necessary to work with more significant amounts of biological material and concentrate the sample in the sample preparation process. When selecting a sample preparation procedure, we compared the recovery results obtained from 11 different procedures, calculated as the ratio of the peak area obtained from analysis of saliva samples spiked before and after the sample preparation procedure (Figure 2).

In the first eight procedures, liquid-liquid extraction was used with different extractants and in the jomb-41-4-2204466T_g00rest - salting-out assisted liquid extraction (SALLE) with different salts. In four of the procedures, saliva was acidified with 0.1% formic acid. In three, it was diluted with deionized water. In the rest, the biological material was subjected to extraction without dilution. We started with sample acidification regarding the information for pKa of 8-isoPGF2α, which is about 4.4 [29]. Before the liquid-liquid extraction, we preextracted with hexane to purify the sample of other non-polar compounds that might interfere with the analyte of interest. Analysis of the separated supernatant showed no detectable amounts of 8-isoPGF2α. Subsequent double extractions were performed with 3% hexane in 1-butanol, 3% hexane in ethyl ethanoate, and 100% ethyl ethanoate. Preliminary laboratory experiments comparing different amounts of hexane (1%, 3%, 5%, or 10%) showed the best results when using 3% hexane as a modifier of the main extractant. The first three procedures achieved a similar recovery of 46%-49% with a similar recovery. The saliva samples were diluted with deionized water in the following three procedures. They were extracted directly with three different extractants. A significantly higher recovery was achieved when using 1-butanol (62.7%) compared to the use of 3% hexane in 1-butanol (44.3%) and 10% ethanol in 1-butanol (42.5%). Liquid-liquid extraction with methanol: chloroform (2:1, v/v) on pre-acidified saliva samples with 0.1% formic acid showed low recovery. The result was similar to the procedure in which the saliva sample was diluted with acetonitrile, then evaporated to a residual volume of 1 mL and double extracted with ethyl ethanoate. Subsequently tested procedures were variants of SALLE, using MgSO_4_, ZnSO_4_, or NaCl. According to the calculated data for the recovery, the procedure of sample preparation 11 was selected. Representative SRM (selective reaction monitoring) chromatograms for the control samples obtained with the selected sample preparation procedure (procedure 11) are shown in Figure 1.

**Figure 3:**
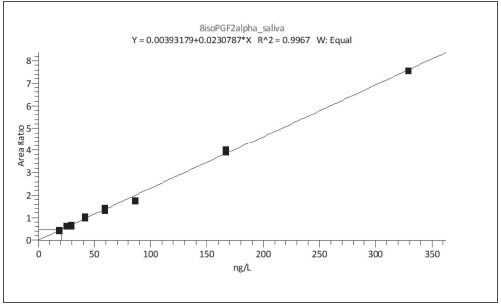
Calibration curve of 8-isoPGF2α

To achieve maximum sensitivity and specificity of the method, considering the low isoprostanes concentration in saliva (in ng/L range), we had to accomplish a very good chromatographic separation and optimal mass detector settings. Our previous experience with the core shell chromatographic column in determining isoprostanes in blood plasma [30] predetermined the use of the same column. Using a gradient elution, we achieved the cleanest and most symmetrical peaks, which was necessary for establishing a very good low limit of detection (LOD) and low limit of quantification (LLOQ) values, respectively 10 ng/L and 25 ng/L. The resulting calibration curve is linear in the range of 25-329 ng/L with R2 larger than 0.995 (Figure 3).

Accuracy and precision were calculated from the results obtained from the analysis of the prepared control samples at four concentration levels. Five control samples from each level in consecutive order were analyzed for within-run calculation. Two samples from each level in four consecutive days were ana-lyzed for between-run calculation. Accuracy and precision completely met the acceptance criteria (Table 1).

**Table 1:** Calculated accuracy and precision at four concentration levels.

Level ng/L	Accuracy (% from theoretical)				Precision	
	within-run		between-run		within-run	between-run
	min	max	min	max		
25	97.3%	108.4%	97.3%	113.9%	4.4%	5.3%
30	98.0%	106.6%	89.7%	109.3%	3.1%	5.4%
55	94.7%	101.1%	94.7%	103.1%	2.7%	2.9%
255	96.7%	102.0%	96.4%	103.1%	2.3%	2.4%

Biological samples contain a wide variety of polarity substances, often coeluting with the desired analyte, which cannot always be cleared in the sample preparation process. The LC-MS/MS methods depend to a large extent on the degree of ionization of the measured analyte and on the various positive or negative effects that other components of the sample may have on it. To compensate for this effect, we use a stable isotope-labeled internal standard (8-iso-PGF2α-d4). The calculated normalized matrix effect was within the allowable, between 89.7% and 113.5% (Table 2).

**Table 2:** Calculated matrix effect. ME – calculated matrix effect for 8-isoPGF2α<br>ME IS – calculated matrix effect for 8-isoPGF2α-d4

Probe	ME	ME IS	Relative ME
SpAf 1	88.1%	87.6%	100.6%
SpAf 2	52.2%	49.1%	106.4%
SpAf 3	92.6%	86.6%	106.9%
SpAf 4	78.3%	80.5%	97.2%
SpAf 5	102.7%	90.4%	113.5%
SpAf 6	84.8%	77.0%	110.1%
SpAf 7	95.1%	99.5%	95.5%
SpAf 8	90.9%	89.4%	101.6%
SpAf 9	97.8%	95.4%	102.5%
SpAf 10	93.6%	97.1%	96.4%
SpAf 11	85.5%	95.3%	89.7%
SpAf 12	84.1%	83.6%	100.5%

The stability of the spiked samples during three freeze-thaw cycles, each of which for 24 hours was between 89.7% and 106.6%. The post-preparative stability for 12 and 24 hours at 4-8°C was in the range of 102.6%-113.2%.

## Conclusion

This study presents a newly developed and validated LS-MS/MS method for the determination of 8-iso-PGF2α in human saliva. The proposed method is based on salting-out assisted liquid-liquid extraction; it is simple and shows a sufficient level of sensitivity and satisfactory accuracy and precision. The method allows quantifying 25.0-329 ng/L of salivary 8-isoPGF2α using only 1 mL of sample.

## Dodatak

### Acknowledgments

This study was funded by the Medical University of Plovdiv, Bulgaria [Grant NO- 01/2020].

### Conflict of interest statement

All the authors declare that they have no conflict of interest in this work.
